# Distribution and Determinants of Vitamin D-Binding Protein, Total, “Non-Bioavailable”, Bioavailable, and Free 25-Hydroxyvitamin D Concentrations among Older Adults

**DOI:** 10.3390/nu13113982

**Published:** 2021-11-09

**Authors:** Anna Zhu, Sabine Kuznia, Tobias Niedermaier, Bernd Holleczek, Ben Schöttker, Hermann Brenner

**Affiliations:** 1 Division of Clinical Epidemiology and Aging Research, German Cancer Research Center (DKFZ), 69120 Heidelberg, Germany; anna.zhu@dkfz-heidelberg.de (A.Z.); s.kuznia@dkfz-heidelberg.de (S.K.); t.niedermaier@dkfz-heidelberg.de (T.N.); b.schoettker@dkfz-heidelberg.de (B.S.); 2 Medical Faculty Heidelberg, Heidelberg University, 69120 Heidelberg, Germany; 3 Saarland Cancer Registry, 66119 Saarbrücken, Germany; b.holleczek@gbe-ekr.saarland.de; 4 Network Aging Research, Heidelberg University, 69115 Heidelberg, Germany; 5 Division of Preventive Oncology, German Cancer Research Center (DKFZ) and National Center for Tumor Diseases (NCT), 69120 Heidelberg, Germany; 6 German Cancer Consortium (DKTK), German Cancer Research Center (DKFZ), 69120 Heidelberg, Germany

**Keywords:** vitamin D-binding protein, bioavailable 25(OH)D, free 25(OH)D, determinants

## Abstract

Background: serum 25-hydroxyvitamin D (25(OH)D) (“total 25 OH(D)”) is the most commonly used indicator of vitamin D status. However, 25(OH)D is mostly bound to the vitamin D binding protein (VDBP) or albumin in blood, and it has been suggested that the remaining bioavailable or free 25(OH)D may be more relevant for vitamin D associated health outcomes. We aimed to explore distributions and determinants of VDBP, total, bioavailable, complementary “non-bioavailable”, and free 25(OH)D in a large cohort of older adults in Germany. Methods: total 25(OH)D, VDBP, and albumin concentrations were measured in blood samples of 5899 men and women aged 50–75 years and used to calculate bioavailable (and complementary “non-bioavailable”) and free 25(OH)D concentrations. Linear regression models were used to evaluate associations of potential determinants of the various vitamin D biomarkers. Results: mean concentrations of VDBP, total, non-bioavailable, bioavailable, and free 25(OH)D were 323.6 µg/mL, 49.8 nmol/L, 43.4 nmol/L, 2.5 ng/mL, and 5.7 pg/mL, respectively. Seasonal variations were observed for all markers, with peak values in spring for VDBP and in summer for total, non-bioavailable, bioavailable, and free 25(OH)D. Consistent inverse associations were seen with age and body mass index for all markers, but divergent associations were seen with C-reactive protein. Strong variations by VDBP genotypes were seen for bioavailable and free 25(OH)D, and, in opposite direction for non-bioavailable 25(OH)D. Conclusion: commonalities and differences in determinants of various markers of vitamin D status were observed, which may help to enable a better understanding of their potential role for various vitamin D related health outcomes.

## 1. Introduction

Vitamin D deficiency is common and closely related to various health outcomes among older adults [1]. The most widely used indicator of vitamin D status is serum 25-hydroxyvitamin D (25(OH)D). It has been suggested, however, that other vitamin D biomarkers may be more relevant with respect to health outcomes [2]. About 85–90% of serum 25(OH)D are bound to vitamin D-binding protein (VDBP) [3]. Serum 25(OH)D that is not bound to VDBP is known as bioavailable 25(OH)D. Around 10–15% of serum 25(OH)D are loosely bound to albumin, and less than 1% is in a free form, known as free 25(OH)D [4,5].

Although a number of studies have addressed distribution and potential determinants of the various vitamin D biomarkers in different populations [2,3,6,7,8], evidence from large-scale epidemiological studies among older adults has remained sparse, mostly descriptive without multivariate adjustments [3,8], or limited to single specific vitamin D biomarkers [7]. The aim of this study was to assess distributions and determinants of VDBP, total, bioavailable (and complementary “non-bioavailable”), and free 25(OH)D concentrations in a large population cohort of older adults in Germany.

## 2. Materials and Methods

### 2.1. Study Design and Population

Our analysis is based on baseline data from the ESTHER study (German: Epidemiologische Studie zu Chancen der Verhütung, Früherkennung und optimierten Therapie chronischer Erkrankungen in der älteren Bevölkerung), a population-based cohort study of older adults conducted statewide in Saarland, a federal state in Southwestern Germany. Details of the study design have been reported elsewhere [9]. Briefly, 9940 men and women aged 50–75 years were recruited by over 400 general practitioners between 2000 and 2002 during a routine health checkup offered in the German healthcare system. The ESTHER study population has been found to be representative of the German older population with respect to sociodemographic characteristics, lifestyle factors, and health conditions [10]. The current analysis was restricted to 5899 participants recruited in 2001 and 2002 for whom measurements of total 25(OH)D, VDBP, genetic markers, and the necessary covariates needed to derive bioavailable and free 25(OH)D were available (for details see below).

### 2.2. Data and Blood Sample Collection

We collected information on covariates including age, sex, education, smoking status, physical activity, intake of multivitamin supplements, and fish consumption by using a comprehensive questionnaire at baseline. General practitioners assessed height, weight, and systolic blood pressure, and provided information on presence of hypertension, diabetes, chronic kidney diseases (CKD), and cardiovascular diseases (CVD) at the time of the health check-up. For our analyses, history of CVD was defined as history of coronary heart disease, myocardial infarction, stroke, or stent/balloon catheter operation for revascularization of coronary arteries. We obtained information on previous incident malignant cancer (excluding non-melanoma skin cancer) through record linkage with the long-standing statewide Saarland Cancer Registry.

Blood samples were collected at baseline by general practitioners, centrifuged, shipped to the study center, and stored at −80 °C until analysis. Baseline laboratory analyses included total cholesterol (measured by enzymatic chromatography), C-reactive protein (CRP, measured by turbidimetry), and albumin (measured by fluorescence immunoassay).

### 2.3. VDBP, Total, Non-Bioavailable, Bioavailable, and Free 25(OH)D Measurements

From the blood samples collected at baseline, we further measured concentrations of total 25(OH)D and VDBP. Total 25(OH)D was measured as the sum of 25(OH)D_2_ and 25(OH)D_3_ in the context of two different research projects for women and men. In a first project among women conducted in 2006, total 25(OH)D concentrations were measured by using the DiaSorin–Liaison analyzer (DiaSorin Inc., Stillwater, MN, USA). In a subsequent project among men conducted in 2009, the DiaSorin–Liaison method was no longer available, and total 25(OH)D was measured using IDS-iSYS (Immunodiagnostic Systems GmbH, Frankfurt Main, Germany). Later both immunoassays were standardized to the gold standard method of liquid chromatography tandem mass spectrometry. Details of the laboratory methods for measuring total 25(OH)D and their standardization have been reported elsewhere [11] and are summarized in the Supplementary File S1. In 2019, VDBP concentrations were measured by enzyme immunoassay (Immundiagnostik Inc., Bensheim, Germany). The intra-assay and inter-assay coefficients of variations were less than 10%. In addition, we extracted genetic data of single nucleotide polymorphisms (SNPs) rs7041 and rs4588 for coding VDBP genotypes from genome-wide genotyping of DNA from whole blood samples using the Illumina Infinium OncoArray and Global Screening Array BeadChip (Illumina, San Diego, CA, USA). More details on genotyping procedures including quality control and imputation have been reported elsewhere [12] and are summarized in the Supplementary File S1.

Bioavailable and free 25(OH)D concentrations were calculated based on the levels of total 25(OH)D, VDBP, albumin, and their affinity constants derived from the VDBP genotypes [13,14]. We used the following equation for calculating free 25(OH)D concentrations:

$$D_{\mathrm{free}}=(-b+\sqrt{b^{2}-4ac})÷2a$$

where a = K_VDBP_ · K_alb_ · D_alb_ + K_VDBP_; b = K_VDBP_ · D_VDBP_ − K_VDBP_ · D_total_ + K_alb_ · D_alb_ + 1; c = −D_total_, and D_free_ indicates free 25(OH)D concentrations; D_alb_ indicates albumin concentrations; D_total_ indicates total 25(OH)D concentrations; D_VDBP_ indicates VDBP concentrations; K_alb_ is the affinity constant between vitamin D and albumin (K_alb_ = 6 × 10^5^ M^−1^); K_VDBP_ is the affinity constant between vitamin D and VDBP (K_VDBP_ = 1.12 × 10^9^ M^−1^ for GC1f-1f; K_VDBP_ = 8.6 × 10^8^ M^−1^ for GC1f-1s; K_VDBP_ = 7.4 × 10^8^ M^−1^ for GC1f-2; K_VDBP_ = 6.0 × 10^8^ M^−1^ for GC1s-1s; K_VDBP_ = 4.8 × 10^8^ M^−1^ for GC1s-2; K_VDBP_ = 3.6 × 10^8^ M^−1^ for GC2-2). All concentrations are expressed in mol/L in calculating equations.

We calculated bioavailable 25(OH)D concentrations by using the following equation:

D_bioavailable_ = D_free_ + D_alb_ = (K_alb_ · D_alb_ + 1) · D_free_

where D_alb_ indicates albumin concentrations; D_bioavailable_ indicates bioavailable 25(OH)D concentrations; D_free_ indicates free 25(OH)D concentrations; K_alb_ is the affinity constant between vitamin D and albumin (K_alb_ = 6 × 10^5^ M^−1^). All concentrations are expressed in mol/L in calculating equations.

We further calculated “non-bioavailable 25(OH)D” concentrations as the difference between total and bioavailable 25(OH)D concentrations.

### 2.4. Statistical Analysis

We summarized the baseline characteristics by using descriptive statistics. Additionally, we visualized the distributions of VDBP, total, non-bioavailable, bioavailable, and free 25(OH)D concentrations by sex. We plotted histograms and density curves to show the general distribution of these vitamin D biomarkers, and further plotted bar charts to assess potential variations by season and month of blood draw, and VDBP genotypes. We also calculated Spearman’s rank correlation coefficients and plotted the correlation analysis of VDBP, total, non-bioavailable, bioavailable, and free 25(OH)D.

We report mean concentrations of VDBP, total, non-bioavailable, bioavailable, and free 25(OH)D by various demographic, behavioral, and medical characteristics. We used linear regression models to assess individual and independent associations of these factors with vitamin D biomarkers. Using linear regression was judged to be appropriate because all outcomes were approximately normally distributed despite some right skewedness for total and non-bioavailable 25(OH)D among females. Overall, 783 participants had at least one missing value in one or more of the included covariates (13.3%). To minimize potential bias, we applied multiple-imputation and report pooled regression results from 20 imputed databases. All analyses were carried out by R software (version: 3.6.2, R Core Team, R Foundation for Statistical Computing, Vienna, Austria). Statistical significance was defined by *p* < 0.05 in two-sided testing.

### 2.5. Ethics Statement

The ESTHER study was approved by the Ethics Committees of the Medical Faculty of the University of Heidelberg and of the Physicians’ Board of Saarland. All participants gave written informed consent.

## 3. Results

Table 1 shows the baseline characteristics of 5899 included participants. Mean age was 62.3 (standard deviation (SD): 6.6) years, and 43.9% were males. Approximately half (50.7%) were never smokers, 32.0%, 14.4%, and 67.0% reported moderate or high physical activity, regular intake of multivitamin supplements, and fish consumption at least once per week, respectively. History of hypertension, diabetes, CVD, cancer, and CKD, were reported for 43.3%, 15.0%, 19.7%, 6.3%, and 8.3% of participants, respectively. The mean body mass index (BMI), systolic blood pressure, total cholesterol, and CRP was 27.7 (SD: 4.4) kg/m^2^, 139.9 (SD: 19.6) mmHg, 230.9 (SD: 42.2) mg/dL, and 4.2 (SD: 8.1) mg/L, respectively.

**Figure 1:**
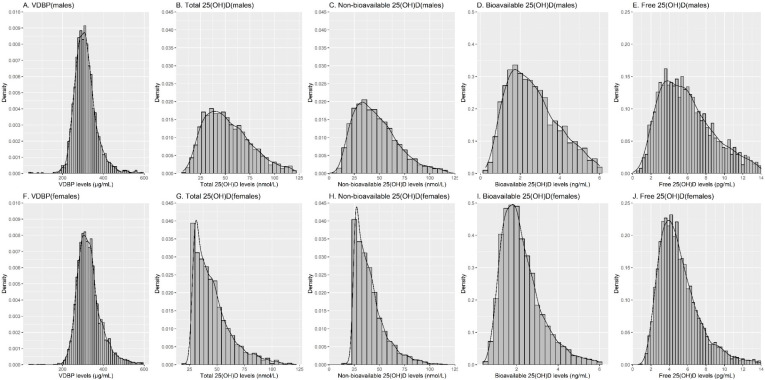
Histograms and density curves of VDBP, total, non-bioavailable, bioavailable, and free 25(OH)D concentrations by sex. Abbreviations: VDBP: vitamin D binding protein.

Overall mean concentrations of VDBP, total, non-bioavailable, bioavailable, and free 25(OH)D were 323.6 µg/mL, 49.8 nmol/L, 43.4 nmol/L, 2.5 ng/mL, and 5.7 pg/mL, respectively. Figure 1 shows distribution plots of VDBP, total, non-bioavailable, bioavailable, and free 25(OH)D concentrations by sex. For VDBP, a close to normal distribution was observed; the distribution of the other vitamin D biomarkers was right-skewed to a moderate extent.

**Figure 2:**
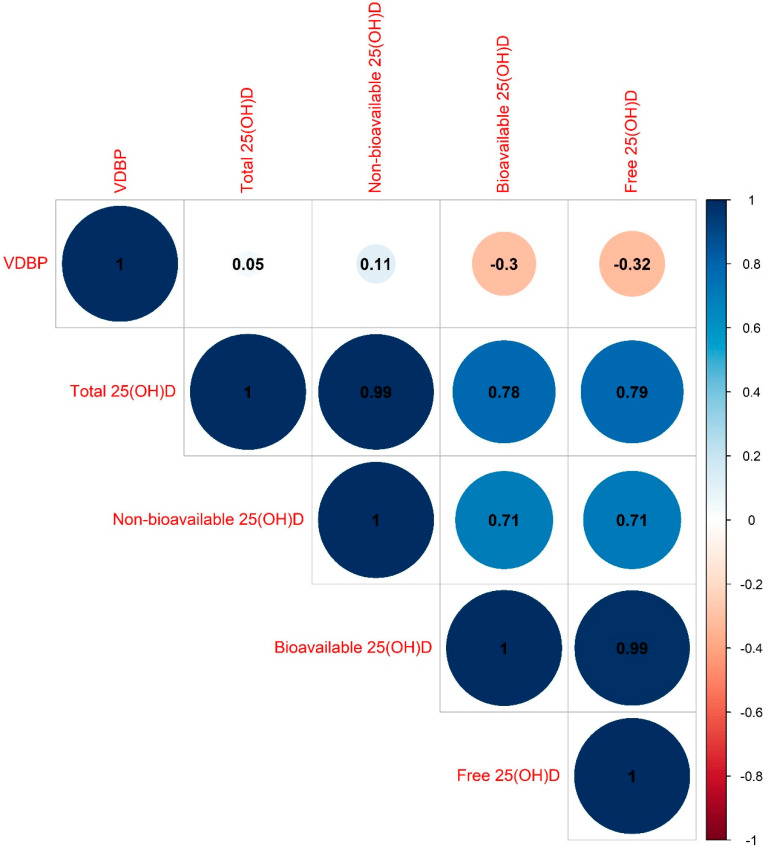
Correlation matrix between VDBP, total, non-bioavailable, bioavailable, and free 25(OH)D concentrations. Abbreviations: VDBP: vitamin D binding protein.

Figure 2 presents the correlation matrix for VDBP, total, non-bioavailable, bioavailable, and free 25(OH)D. VDBP concentrations were weakly correlated with total and non-bioavailable 25(OH)D, and moderate negative correlations with bioavailable and free 25(OH)D concentrations were observed (Spearman’s rank correlation coefficients: 0.05, 0.11, −0.30, and −0.32, respectively). Total 25(OH)D concentrations were highly correlated with non-bioavailable, bioavailable, and free 25(OH)D concentrations (Spearman’s rank correlation coefficients were 0.99, 0.78, and 0.79, respectively). Bioavailable 25(OH)D levels were also strongly correlated with free 25(OH)D levels (Spearman’s rank correlation coefficient: 0.99).

**Figure 3:**
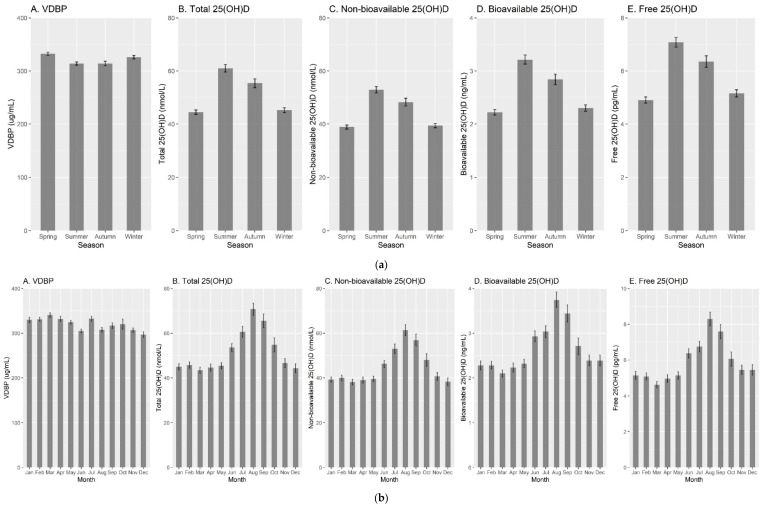

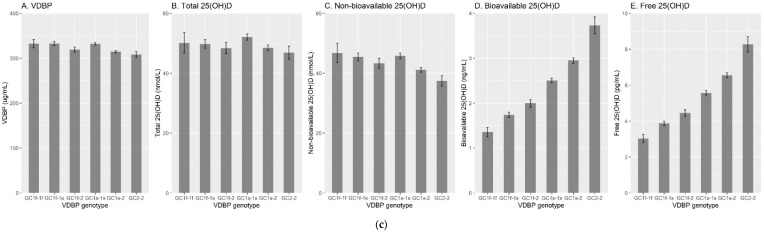
VDBP, total, non-bioavailable, bioavailable, and free 25(OH)D concentrations by season and month of blood draw, and VDBP genotype. (**a**) By season of blood draw (spring: March, April, and May; summer: June, July, and August; autumn: September, October, and November; winter: December, January, and February). (**b**) By month of blood draw. (**c**) By VDBP genotype. Abbreviations: VDBP: vitamin D binding protein.

Figure 3 further plots the variations in VDBP, total, non-bioavailable, bioavailable, and free 25(OH)D concentrations by season and month of blood draw, and VDBP genotype. Seasonal variations were observed for all markers, with peak values in spring for VDBP (mean: 332.3 µg/mL) and in summer for total, non-bioavailable, bioavailable, and free 25(OH)D (mean: 61.0 nmol/L, 52.9 nmol/L, 3.2 ng/mL, and 7.1 pg/mL, respectively). Additionally, the concentrations of vitamin D biomarkers differed by VDBP genotype. Participants with GC2-2 genotype had the lowest levels of VDBP (mean: 308.0 µg/mL), total (mean: 46.8 nmol/L), and non-bioavailable 25(OH)D (mean: 37.4 nmol/L), but had the highest levels of bioavailable (mean: 3.7 ng/mL), and free 25(OH)D (mean: 8.3 pg/mL). Participants with GC1f-1f genotype had the lowest levels of bioavailable (mean: 1.4 ng/mL), and free 25(OH)D (mean: 3.0 pg/mL), but the highest level of non-bioavailable 25(OH)D (mean: 46.7 nmol/L), and the second highest level of VDBP (mean: 332.4 µg/mL), which was very close to those with GC1s-1s genotype (mean: 332.0 µg/mL) and GC1f-1s genotype (mean: 332.6 µg/mL). Those with GC1s-1s genotype had the highest level of total 25(OH)D (mean: 52.1 nmol/L).

Table 2 presents VDBP, total, non-bioavailable, bioavailable, and free 25(OH)D concentrations by selected participant characteristics, and Table 3 presents results of the corresponding regression models in which all of the covariates were mutually adjusted for (in addition to adjustment by VDBP genotype and season of blood draw). VDBP levels decreased with increasing age and increasing BMI, and were lower in men and those with diabetes. By contrast, higher levels were observed among those reporting regular intake of vitamin supplements, with high total cholesterol and CRP levels. Both total and non-bioavailable 25(OH)D concentrations decreased with increasing age and increasing BMI, and were lower among current smokers, and those with diabetes. However, higher concentrations were seen among males, those reporting more physical activity and regular intake of vitamin supplements. Non-bioavailable 25(OH)D concentrations were also higher among those with higher CRP levels. Bioavailable and free 25(OH)D concentrations decreased with increasing age and increasing BMI, and were lower among current smokers and those with high levels of total cholesterol. Lower bioavailable 25(OH)D levels were further seen among those with higher CRP levels. Higher concentrations of bioavailable and free 25(OH)D levels were seen among males, those reporting more physical activity and regular intake of vitamin supplements.

## 4. Discussion

In this large population-based cohort of older adults from Germany, total 25(OH)D levels were found to be low (mean: 49.8 nmol/L), and to strongly vary by season of blood draw. Levels of non-bioavailable, bioavailable, and free 25(OH)D were strongly correlated with total 25(OH)D levels and showed similar major seasonal variation, with the highest concentrations in summer, and the lowest concentrations in spring. Despite strongly different absolute concentrations, all 25(OH)D measures also showed similar associations with potential non-genetic determinants, with decreasing levels with increasing age and BMI, and higher levels among males, non-smokers, and those reporting regular multivitamin intake. By contrast, VDBP genotypes showed opposite associations with bioavailable and free 25(OH)D (lowest levels among those with GC1f-1f genotype, highest among those with GC2-2 genotype), and non-bioavailable 25(OH)D (lowest among those with GC2-2 genotype, highest among those with GC1f-1f genotype). VDBP concentrations were highest in spring, lower in men than in women and unrelated to smoking, but they also strongly decreased with increasing age and increasing BMI. They showed moderate inverse correlations with bioavailable and free 25(OH)D. In addition, some distinct patterns were seen, with markedly higher VDBP concentrations among those with high cholesterol and high CRP concentrations.

The associations of total 25(OH)D with potential determinants observed in our study are consistent with those observed in multiple previous studies and are meanwhile well established. This particularly applies to the strong seasonal variation caused by seasonal variation in ultraviolet-B exposure [15,16], which is insufficient for effective synthesis of cholecalciferol in the skin during the winter months in Germany. Likewise, the strong decrease of 25(OH)D levels with increasing age as a result of decreasing ability of the skin to synthesize cholecalciferol and potentially less exposure to ultraviolet-B radiation is well established [9,17]. By contrast, evidence on potential determinants of specific components of 25(OH)D has been rather sparse and partly conflicting [2,8,18,19]. To our knowledge, ours is the first study to comprehensively assess associations of potential determinants with various components, including non-bioavailable, bioavailable, and free 25(OH)D. The high positive correlations between those measures (and in particular between each of them and total 25(OH)D) and the consistent patterns of associations with potential determinants (apart from genotypes) do not support suggestions for the need to evaluate different 25(OH)D components for assessing vitamin D status in clinical practice in an ethnically homogeneous population, such as our cohort, in which the vast majority of participants were Caucasians. Nevertheless, given the strong variation of bioavailable and free 25(OH)D by VDBP genotypes, and the strong variation of VDBP genotypes between ethnic groups [13,14,20], different patterns might be expected and the role of different 25(OH)D components may be different in ethnically more diverse populations. Further research should address the associations of various 25(OH)D components with clinical outcomes in longitudinal studies in different types of populations.

The strong genetic determination of VDBP concentrations is well established [13,20]. Subjects with GC2-2 genotype have significantly lower VDBP concentrations than those with any other genotypes. In agreement with several previous studies [21,22], we found those with GC1f-1f genotype to have the highest VDBP concentrations. Different patterns were seen in the study by Powe et al. [13], where GC1f-1f was the most frequent genotype among Black Americans and associated with the lowest VDBP levels. Possible reasons for this apparent discrepancy, such as differences in antibodies used in the VDBP assays, or other factors and their possible clinical relevance, should be addressed in further research.

To our knowledge, our study is the first to comprehensively address potential non-genetic determinants of VDBP levels. Despite their strong genetic determination, quite pronounced inverse associations of VDBP levels were also seen with age and BMI, similarly to those seen for 25(OH)D concentrations. In contrast to the latter, however, VDBP concentrations were higher in women, and in participants with high cholesterol and CRP levels. The reasons for these strong associations are unclear and require further study. One possible explanation for the association observed with CRP levels might be an increase of VDBP concentrations in response to inflammation [23]. Our large study also disclosed small seasonal variations with peak values in spring in VDBP concentrations, which may previously not have been detected due to sample size limitations [15].

Our study has several strengths. To our knowledge, our study is the first one to provide a parallel comprehensive comparison of determinants of VDBP, total, bioavailable, and free 25(OH)D concentrations. Our study also has a much larger sample size than most previous studies assessing these various vitamin D related parameters. In addition to a range of genetic, lifestyle, and dietary factors, we simultaneously considered various health conditions reported by physicians in multivariable analyses. Furthermore, due to the population-based nature of our cohort, the generalizability of our findings for the older population may be higher than in studies focusing on specific patient groups.

Our study also has a number of limitations. Our analysis was limited to cross-sectional data, which limits any derivation of temporal or causal relationships. Although we have considered a large number of covariates in multivariable analyses, residual confounding might not be ruled out due to the observational nature of the study. We did not directly measure bioavailable and free 25(OH)D, but determined it from total 25(OH)D, VDBP, and albumin concentrations by previously suggested equations. Measured concentrations of free 25(OH)D have been reported to be lower than the calculated ones, especially under physiologic and pathologic conditions (15). Despite these limitations, the quite distinct associations and patterns observed in our study may stimulate further research towards a more complete understanding of the determinants and health relevance of VDBP and the various components of 25(OH)D.

## 5. Conclusions

In this large population-based study of older adults from Germany, we found high correlations of total 25(OH)D with bioavailable, free, and non-bioavailable 25(OH)D, along with quite consistent associations of these markers of vitamin D status with potential non-genetic determinants, despite quite diverse associations with VDBP genotypes. In addition, apart from their genetic determination, VDBP concentrations were found to strongly vary by a number of non-genetic potential determinants. The biological mechanisms and clinical implications of the observed patterns deserve careful elucidation in further research to better understand a potential role of vitamin D in its various compartments for health at older age.

## Figures and Tables

**Table 1:** Baseline characteristics of the study participants (N = 5899).

Characteristic	Mean (SD)	N (%)
Age (years)	62.3 (6.6)	
<60		1972 (33.4)
60–64		1589 (26.9)
65–69		1392 (23.6)
≥70		946 (16.0)
Sex, male		2589 (43.9)
School education		
≤9 years		4298 (74.8)
10–11 years		800 (13.9)
≥12 years		645 (11.2)
Smoking status		
Never smokers		2904 (50.7)
Former smokers		1871 (32.7)
Current smokers		949 (16.6)
Moderate/high physical activity		1883 (32.0)
Regular intake of multivitamin supplements		831 (14.4)
Fish consumption (≥1 time/week)		3714 (67.0)
Hypertension		2545 (43.3)
Diabetes		873 (15.0)
Cardiovascular disease		1161 (19.7)
Cancer		370 (6.3)
Chronic kidney disease		491 (8.3)
Body mass index (kg/m^2^)	27.7 (4.4)	
Systolic blood pressure (mm Hg)	139.9 (19.6)	
Total cholesterol (mg/dL)	230.9 (42.2)	
C-reactive protein (mg/L)	4.2 (8.1)	

N (%) refers to the original data without imputation. Abbreviations: SD: standard deviation.

**Table 2:** VDBP, total, non-bioavailable, bioavailable, and free 25(OH)D concentrations by selected characteristics.

Characteristics	Group	N	VDBP (µg/mL)	Total 25(OH)D (nmol/L)	Non-Bioavailable 25(OH)D (nmol/L)	Bioavailable 25(OH)D (ng/mL)	Free 25(OH)D (pg/mL)
			Mean (SE)	Mean (SE)	Mean (SE)	Mean (SE)	Mean (SE)
All participants			323.6 (0.85)	49.8 (0.29)	43.4 (0.26)	2.5 (0.02)	5.7 (0.04)
Age	<60 years	2229	**329.5 (1.43)**	**51.3 (0.52)**	**44.8 (0.46)**	**2.6 (0.03)**	**5.7 (0.07)**
	60–64 years	1664	**324.6 (1.57)**	**51.2 (0.57)**	**44.6 (0.50)**	**2.6 (0.03)**	**5.8 (0.07)**
	65–69 years	1267	**320.1 (1.72)**	**48.4 (0.56)**	**42.2 (0.49)**	**2.5 (0.04)**	**5.6 (0.08)**
	≥70 years	739	**314.8 (2.33)**	**46.4 (0.72)**	**40.3 (0.62)**	**2.4 (0.05)**	**5.5 (0.11)**
Sex	Females	3310	**332.0 (1.16)**	**46.1 (0.30)**	**40.3 (0.26)**	**2.3 (0.02)**	**5.1 (0.04)**
	Males	2589	**312.9 (1.20)**	**54.6 (0.53)**	**47.3 (0.46)**	**2.9 (0.03)**	**6.4 (0.07)**
School education	≤ 9 years	4429	324.2 (1.00)	**49.1 (0.33)**	**42.7 (0.29)**	**2.5 (0.02)**	**5.6 (0.05)**
	10–11 years	816	322.9 (2.03)	**51.4 (0.79)**	**44.9 (0.69)**	**2.6 (0.05)**	**5.8 (0.10)**
	≥12 years	654	320.3 (2.44)	**52.8 (1.00)**	**45.9 (0.88)**	**2.8 (0.06)**	**6.1 (0.13)**
Smoking status	Never smokers	3005	**325.1 (1.19)**	**48.7 (0.37)**	**42.6 (0.33)**	**2.5 (0.02)**	**5.5 (0.05)**
	Former smokers	1918	**319.5 (1.52)**	**53.4 (0.56)**	**46.4 (0.49)**	**2.8 (0.04)**	**6.1 (0.08)**
	Current smokers	976	**326.9 (1.94)**	**46.1 (0.72)**	**40.1 (0.64)**	**2.3 (0.04)**	**5.2 (0.10)**
Physical activity	Low	4013	323.5 (0.99)	**47.8 (0.34)**	**41.7 (0.30)**	**2.4 (0.02)**	**5.4 (0.05)**
	Moderate/high	1886	323.8 (1.60)	**54.0 (0.56)**	**47.0 (0.49)**	**2.8 (0.04)**	**6.2 (0.08)**
Regular multivitamin intake	No	5043	**322.4 (0.88)**	**49.4 (0.32)**	**43.0 (0.28)**	**2.5 (0.02)**	**5.6 (0.04)**
	Yes	856	**330.9 (2.66)**	**52.3 (0.77)**	**45.8 (0.68)**	**2.6 (0.05)**	**5.8 (0.10)**
Fish consumption	<1 time/week	1955	322.2 (1.37)	**49.4 (0.53)**	**43.1 (0.46)**	**2.5 (0.03)**	**5.6 (0.07)**
	≥1 time/week	3944	324.3 (1.07)	**50.0 (0.35)**	**43.5 (0.31)**	**2.6 (0.02)**	**5.7 (0.05)**
Hypertension	No	3355	324.9 (1.18)	**50.6 (0.40)**	**44.1 (0.35)**	**2.6 (0.03)**	**5.8 (0.06)**
	Yes	2544	321.8 (1.20)	**48.7 (0.43)**	**42.5 (0.37)**	**2.5 (0.03)**	**5.5 (0.06)**
Diabetes	No	5044	**326.0 (0.93)**	**50.3 (0.32)**	**43.9 (0.28)**	**2.6 (0.02)**	5.7 (0.04)
	Yes	855	**309.3 (1.96)**	**46.7 (0.69)**	**40.7 (0.61)**	**2.4 (0.04)**	5.5 (0.09)
Cardiovascular disease	No	4739	**325.5 (0.95)**	**50.0 (0.32)**	**43.6 (0.28)**	**2.5 (0.02)**	5.7 (0.05)
	Yes	1160	**316.0 (1.85)**	**48.9 (0.68)**	**42.6 (0.60)**	**2.5 (0.04)**	5.7 (0.09)
Cancer	No	5447	323.4 (0.89)	49.8 (0.30)	43.4 (0.27)	2.6 (0.02)	5.7 (0.04)
	Yes	452	325.4 (2.69)	49.3 (1.04)	43.1 (0.92)	2.5 (0.06)	5.5 (0.14)
Chronic kidney disease	No	5405	323.7 (0.88)	**49.9 (0.30)**	**43.5 (0.26)**	**2.6 (0.02)**	**5.7 (0.04)**
	Yes	494	322.2 (3.12)	**48.5 (1.18)**	**42.2 (1.01)**	**2.5 (0.08)**	**5.6 (0.18)**
Body mass index	<25 kg/m^2^	1619	**331.3 (1.68)**	**51.5 (0.58)**	**45.0 (0.52)**	**2.6 (0.03)**	**5.8 (0.08)**
	25–29.9 kg/m^2^	2750	**322.4 (1.23)**	**50.8 (0.44)**	**44.2 (0.39)**	**2.6 (0.03)**	**5.8 (0.06)**
	30–34.9 kg/m^2^	1188	**319.3 (1.86)**	**47.2 (0.57)**	**41.2 (0.50)**	**2.4 (0.04)**	**5.4 (0.08)**
	≥35 kg/m^2^	342	**311.6 (2.84)**	**42.6 (0.85)**	**37.2 (0.75)**	**2.1 (0.06)**	**4.9 (0.13)**
Total cholesterol	<200 mg/dL	1345	**310.5 (1.68)**	**51.8 (0.65)**	**45.0 (0.57)**	**2.7 (0.04)**	**6.1 (0.09)**
	≥200 mg/dL	4554	**327.5 (0.97)**	**49.2 (0.33)**	**42.9 (0.29)**	**2.5 (0.02)**	**5.5 (0.05)**
C-reactive protein	<3 mg/L	3619	**317.7 (1.03)**	**50.6 (0.37)**	**44.0 (0.33)**	**2.6 (0.02)**	**5.8 (0.05)**
	≥3 mg/L	2280	**332.9 (1.43)**	**48.5 (0.47)**	**42.5 (0.42)**	**2.4 (0.03)**	**5.4 (0.07)**

Bold print indicates *p* < 0.05. Abbreviations: SE: standard error; VDBP: vitamin D binding protein.

**Table 3:** Associations of various characteristics with VDBP, total, non-bioavailable, bioavailable, and free 25(OH)D concentrations: results of multiple linear regression.

Characteristics	Group	VDBP	Total 25(OH)D	Non-Bioavailable 25(OH)D	Bioavailable 25(OH)D	Free 25(OH)D
		Estimate (SE)	Estimate (SE)	Estimate (SE)	Estimate (SE)	Estimate (SE)
Age	<60 years	Ref	Ref	Ref	Ref	Ref
	60–64 years	**−4.54 (2.14)**	−0.51 (0.71)	−0.46 (0.62)	−0.02 (0.04)	−0.01 (0.09)
	65–69 years	**−9.03 (2.26)**	**−3.27 (0.74)**	**−3.00 (0.65)**	**−0.11 (0.04)**	−0.16 (0.09)
	≥70 years	**−14.88 (2.61)**	**−5.59 (0.86)**	**−5.10 (0.75)**	**−0.20 (0.05)**	**−0.33 (0.11)**
Sex	Females	Ref	Ref	Ref	Ref	Ref
	Males	**−15.48 (1.86)**	**7.82 (0.61)**	**6.43 (0.53)**	**0.55 (0.04)**	**1.11 (0.08)**
School education	≤9 years	Ref	Ref	Ref	Ref	Ref
	10–11 years	−4.21 (2.43)	1.51 (0.79)	1.26 (0.70)	0.10 (0.05)	0.18 (0.10)
	≥12 years	0.09 (2.68)	−0.77 (0.88)	−0.74 (0.77)	−0.01 (0.05)	−0.08 (0.11)
Smoking status	Never smokers	Ref	Ref	Ref	Ref	Ref
	Former smokers	2.13 (2.04)	1.28 (0.66)	1.12 (0.58)	0.06 (0.04)	**0.16 (0.08)**
	Current smokers	0.05 (2.50)	**−5.54 (0.79)**	**−4.84 (0.70)**	**−0.28 (0.05)**	**−0.57 (0.10)**
Physical activity	Low	Ref	Ref	Ref	Ref	Ref
	Moderate/high	1.98 (1.79)	**3.33 (0.59)**	**2.88 (0.52)**	**0.18 (0.03)**	**0.38 (0.08)**
Regular multivitamin intake	No	Ref	Ref	Ref	Ref	Ref
	Yes	**6.87 (2.37)**	**3.84 (0.78)**	**3.50 (0.68)**	**0.14 (0.05)**	**0.32 (0.10)**
Fish consumption	<1 time/week	Ref	Ref	Ref	Ref	Ref
	≥1 time/week	2.39 (1.80)	0.43 (0.59)	0.42 (0.51)	0.01 (0.03)	0.03 (0.07)
Hypertension	No	Ref	Ref	Ref	Ref	Ref
	Yes	0.99 (1.71)	−0.31 (0.57)	−0.25 (0.50)	−0.02 (0.03)	−0.10 (0.07)
Diabetes	No	Ref	Ref	Ref	Ref	Ref
	Yes	**−10.84 (2.40)**	**−2.40 (0.79)**	**−2.22 (0.69)**	−0.07 (0.05)	−0.17 (0.10)
Cardiovascular disease	No	Ref	Ref	Ref	Ref	Ref
	Yes	−1.29 (2.16)	−0.85 (0.71)	−0.74 (0.62)	−0.04 (0.04)	−0.10 (0.09)
Cancer	No	Ref	Ref	Ref	Ref	Ref
	Yes	2.85 (3.05)	−0.18 (1.01)	−0.10 (0.88)	−0.03 (0.06)	−0.08 (0.13)
Chronic kidney disease	No	Ref	Ref	Ref	Ref	Ref
	Yes	4.59 (3.04)	0.75 (1.01)	0.67 (0.88)	0.03 (0.06)	0.11 (0.13)
Body mass index	<25 kg/m^2^	Ref	Ref	Ref	Ref	Ref
	25–29.9 kg/m^2^	**−8.21 (2.01)**	**−1.69 (0.66)**	**−1.60 (0.58)**	−0.04 (0.04)	−0.07 (0.08)
	30–34.9 kg/m^2^	**−13.69 (2.52)**	**−4.63 (0.83)**	**−4.27 (0.73)**	**−0.14 (0.05)**	**−0.31 (0.11)**
	≥35 kg/m^2^	**−26.31 (3.88)**	**−8.92 (1.28)**	**−8.11 (1.12)**	**−0.32 (0.07)**	**−0.61 (0.16)**
Total cholesterol	<200 mg/dL	Ref	Ref	Ref	Ref	Ref
	≥200 mg/dL	**13.19 (1.97)**	−1.22 (0.65)	−1.00 (0.57)	−0.09 (0.04)	**−0.34 (0.08)**
C-reactive protein	<3 mg/L	Ref	Ref	Ref	Ref	Ref
	≥3 mg/L	**17.86 (1.75)**	1.04 (0.58)	**1.27 (0.51)**	**−0.09 (0.03)**	−0.10 (0.07)

Bold print indicates *p* < 0.05. Abbreviations: SE: standard error; VDBP: vitamin D binding protein. The regression models were also adjusted for VDBP genotypes and seasons of blood draw.

## Data Availability

The data presented in this study are available on request from the corresponding author. The data are not publicly available due to privacy or ethical consideration.

## Supplementary Materials

The following are available online at https://www.mdpi.com/article/10.3390/nu13113982/s1, File S1: Measurement and standardization of total 25(OH)D concentrations.
