# Supplementary material for: Distribution and Determinants of Vitamin D-Binding Protein, Total, “Non-Bioavailable”, Bioavailable, and Free 25-Hydroxyvitamin D Concentrations among Older Adults
Source: Nutrients. 2021 Nov 9;13(11):3982. doi: 10.3390/nu13113982 (PMC8624653; doi:10.3390/nu13113982)
Supplement: Supplementary file 1 [file nutrients-13-03982-s001.zip › nutrients-1408702-supplementary.pdf]

Supplementary File

Measurement and standardization of total 25(OH)D concentrations

The automated Diasorin-Liaison analyzer (Diasorin Inc) was used to measure serum 25(OH)D concentrations among women, which was conducted in the central laboratory of the University Clinic of Heidelberg (Heidelberg, Germany) in 2006. The analyzer has a within-assay coefficient of variation (CV) of 8-21% and a between-assay CV of 8-34%. The lowest detection limit was 15 nmol/L. The automated IDS-iSYS analyzer (Immunodiagnostic Systems GmbH) was used for measurements among men in 2009, which was conducted in the laboratory of the Institute for Experimental Endocrinology, Charité University Medicine (Berlin, Germany). The assay has an intraassay CV <7.3%, an interassay CV <8.9%. The lowest detection limit was 9 nmol/L. Both immunoassays were standardized to the gold standard method LC-MS/MS. Baseline serum samples of 100 males and females were randomly extracted from each assay and re-measured by using isotope-dilution LC-MS/MS in 2011 in the Department of Clinical Chemistry, Canisius Wilhelma Hospital (Nijmegen, Netherlands). The human serum calibrator of Chromsystem, Munich, Germany was used to standardize the LC-MS/MS because the NIST SRM standard used nowadays was not available at that time. Three pairs of influential outliers (> 2 standard deviations of the mean assay difference) were excluded for each assay, which resulted in 97 pairs of 25(OH)D values for the standardization of each immunoassay. The Spearman's rank correlation between measurements with the Diasorin-Liaison analyzer and LC-MS/MS, and between the IDS-iSYS analyzer and LC-MS/MS was high (coefficients: 0.83 and 0.86, respectively). Although absolute measurements of the IDS-iSYS analyzer and LC-MS/MS were comparable, values were consistently substantially lower for measurements with the Diasorin-Liaison analyzer than with LC-MS/MS. Therefore, ordinary least-squares linear regression equations were fitted, and results were used for the standardization of 25(OH)D concentrations measured with the Diasorin-Liaison analyzer:

25(OH)D (LC-MS/MS) [nmol/L]=0.7989 * 25(OH)D (Diasorin-Liaison analyzer) [nmol/L] + 17.58 nmol/L

or with the IDS-iSYS analyzer:

25(OH)D (LC-MS/MS) [nmol/L]=0.9526 * 25(OH)D (IDS-iSYS analyzer) [nmol/L] – 0.3222 nmol/L

Genotyping and imputation of SNPs rs7041 and rs4588

Blood samples were taken during the routine health examination and stored at −80 °C until analysis. Deoxyribonucleic acid (DNA) was collected from whole blood samples through a salting out procedure. The extracted DNA was genotyped using the Illumina Infinium OncoArray and Global Screening Array BeadChips (Illumina, San Diego, CA, USA). Genotypes for common variants across the genome were imputed using data from 1000 Genomes Project (phase 3, Oct. 2014) with IMPUTE2 v2.3.2 after pre-phasing with SHAPEIT software v2.12. Poorly imputed SNPs defined by an information metric I < 0.70 were excluded. All genomic locations are given in NCBI Build 37/UCSC hg19 coordinates. The singleNucleotide polymorphisms (SNPs) with a minor allele frequency (MAF) < 1% were excluded. PLINK v1.90 was used to extract the required SNPs. VDBP genotypes were coded by combining SNP rs7041 and SNP rs4588 genotypes (**Table S1**).

Table S1. Combination of SNP rs7041 and rs4588 for coding VDBP genotype

rs7041 genotype	rs4588 genotype	VDBP genotype
TT	CC	GC1f-1f
TG	CC	GC1f-1s
TT	CA	GC1f-2
GG	CC	GC1s-1s
TG	CA	GC1s-2
TT	AA	GC2-2
